# Author Correction: Mapping global lake dynamics reveals the emerging roles of small lakes

**DOI:** 10.1038/s41467-022-34140-9

**Published:** 2022-10-25

**Authors:** Xuehui Pi, Qiuqi Luo, Lian Feng, Yang Xu, Jing Tang, Xiuyu Liang, Enze Ma, Ran Cheng, Rasmus Fensholt, Martin Brandt, Xiaobin Cai, Luke Gibson, Junguo Liu, Chunmiao Zheng, Weifeng Li, Brett A. Bryan

**Affiliations:** 1 School of Environmental Science and Engineering, Southern University of Science and Technology, Shenzhen, China; 2 Department of Urban Planning and Design, The University of Hong Kong, Hong Kong SAR, China; 3 Urban Systems Institute, The University of Hong Kong, Hong Kong SAR, China; 4 Department of Geosciences and Natural Resource Management, University of Copenhagen, Copenhagen, Denmark; 5 Department of Biology, University of Copenhagen, Copenhagen, Denmark; 6 Department of Physical Geography and Ecosystem Science, Lund University, Lund, Sweden; 7 Guangdong Provincial Key Laboratory of Brain-inspired Intelligent Computation, Department of Computer Science and Engineering, Southern University of Science and Technology, Shenzhen, China; 8 Institute of Geodesy and Geophysics, Chinese Academy of Sciences, Wuhan, China; 9 School of Water Conservancy, North China University of Water Resources and Electric Power, 450046 Zhengzhou, China; 10 EIT Institute for Advanced Study, Ningbo, China; 11 Institute for Climate and Carbon Neutrality, The University of Hong Kong, Hong Kong SAR, China; 12 Centre for Integrative Ecology, School of Life and Environmental Sciences, Deakin University, Burwood, Victoria Australia

**Keywords:** Limnology, Hydrology, Limnology, Hydrology

Correction to: *Nature Communications* 10.1038/s41467-022-33239-3, published online 01 October 2022

In this article, the affiliation ‘Department of Physical Geography and Ecosystem Science, Lund University, Lund, Sweden’ for Jing Tang was missing. The original article has been corrected.

