# Supplementary material for: Mapping global lake dynamics reveals the emerging roles of small lakes
Source: Nat Commun. 2022 Oct 1;13:5777. doi: 10.1038/s41467-022-33239-3 (PMC9526744; doi:10.1038/s41467-022-33239-3)
Supplement: Supplementary file 1 — Supplementary Information [file 41467_2022_33239_MOESM1_ESM.docx]

# Supplementary Information for

**Mapping global lake dynamics reveals the emerging roles of small lakes**

Inventory:

Supplementary Figures 1-14

Supplementary Tables 1-5

Supplementary Notes 1-3

Supplementary References

# Supplementary Figures
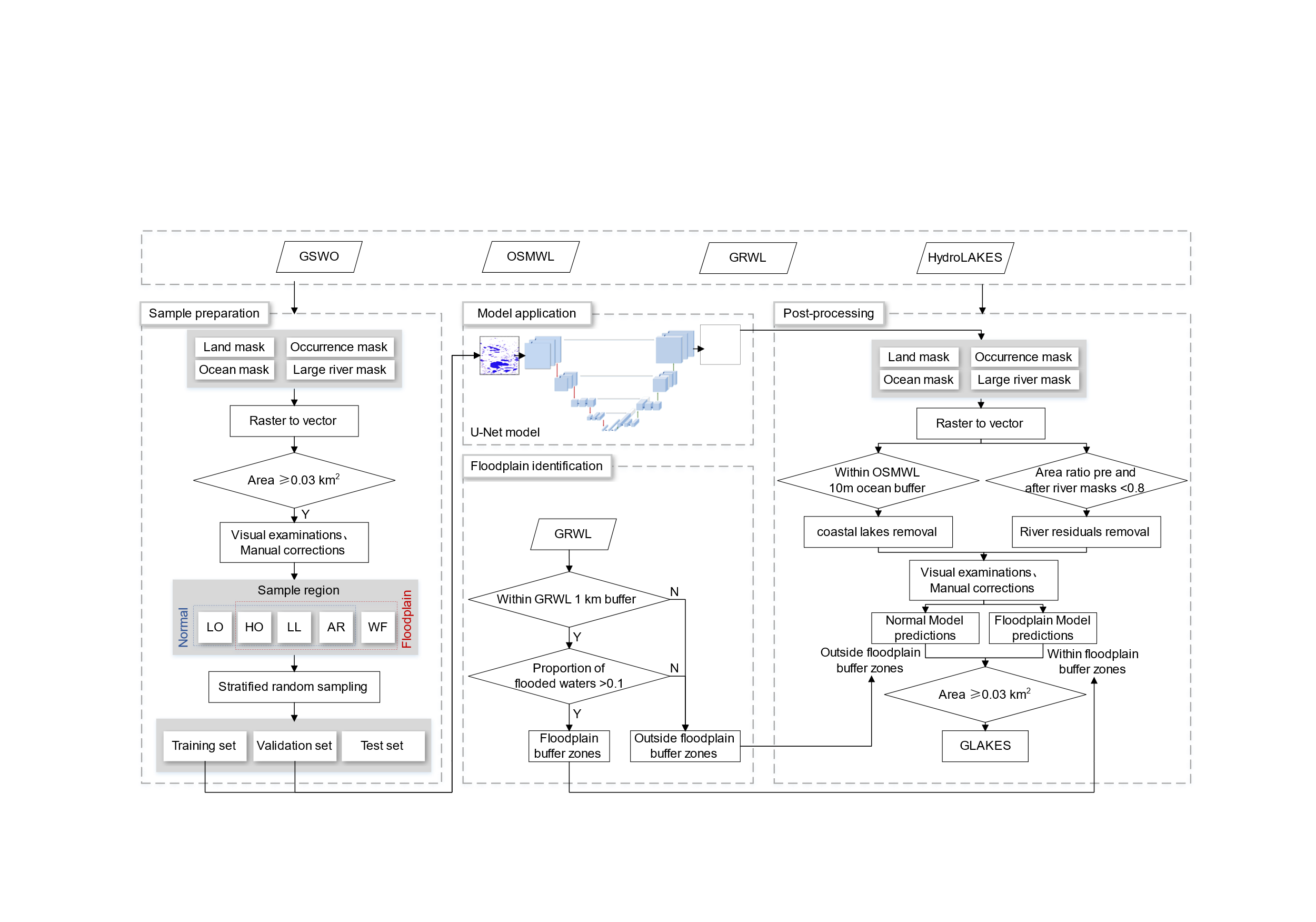


## **Supplementary Figure 1 |** Flowchart for developing the GLAKES dataset.

The complete workflow for the extraction of GLAKES lake polygons can be further divided into the following four modules. 1) Sample preparation: lake samples are generated and allocated to the training, validation and test sets for both Normal Model and Floodplain Model, where different region types representing variated lake features are considered. 2) Model application: the two U-Net models are trained to learn different features of lakes from the GSWO map, and each yield a raw global lake classification map. 3) Floodplain identification: the floodplain buffer zones are determined to combine the outputs from the Floodplain Model and Normal Model. 4) Post-processing: the two global lake classification maps further undergo several post-processing steps and are combined to generate the final GLAKES lake polygons.

**
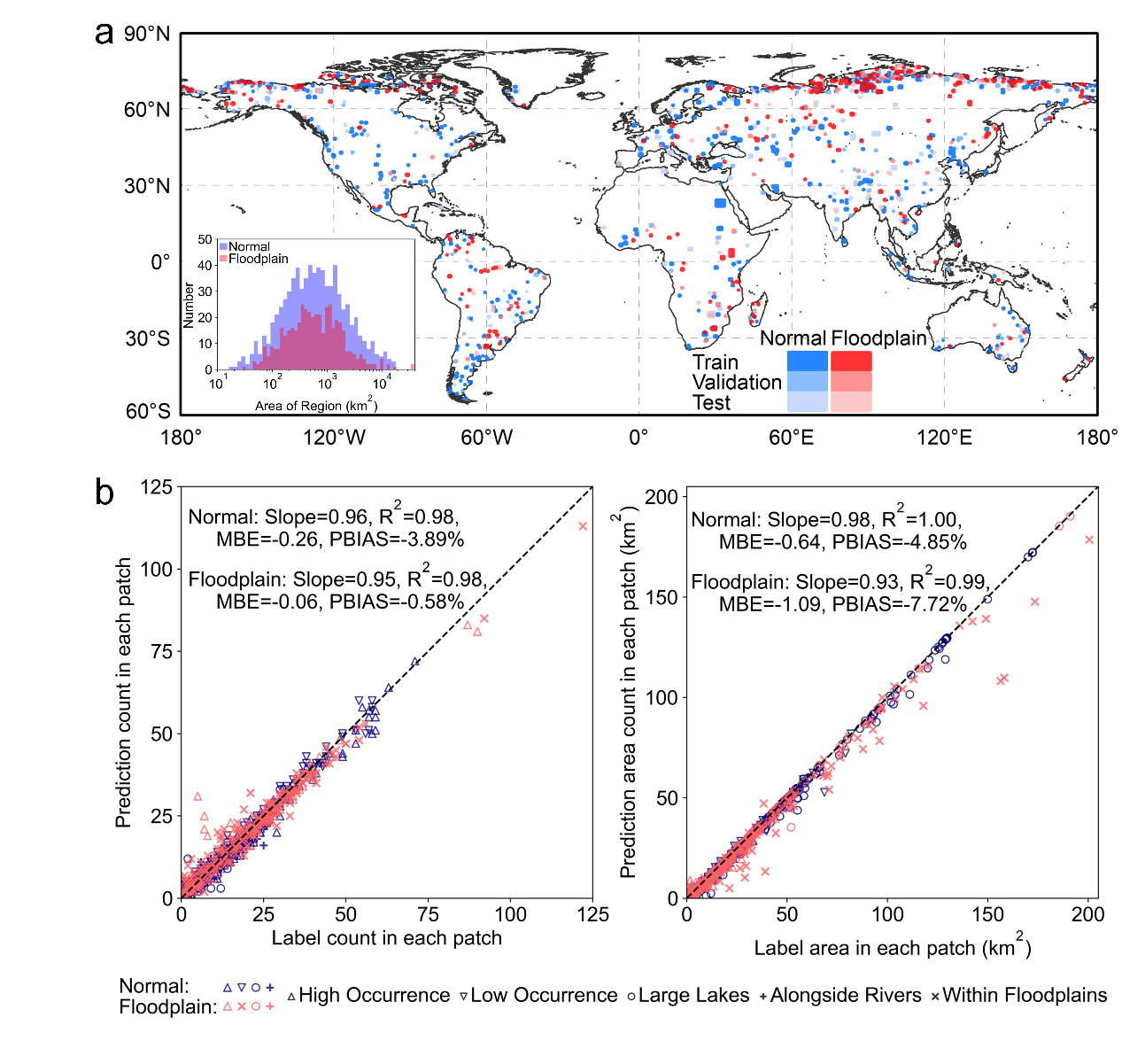
**

## **Supplementary Figure 2 |** Development and validation of the deep-learning algorithm for predicting lake extents.

**a** Spatial distribution of the sample regions selected for training, validation, and testing, along with the size range of the sample regions. **b** The total lake count/area within each patch (512 × 512 pixels) from labels and predictions are compared, where region types are also annotated.

**
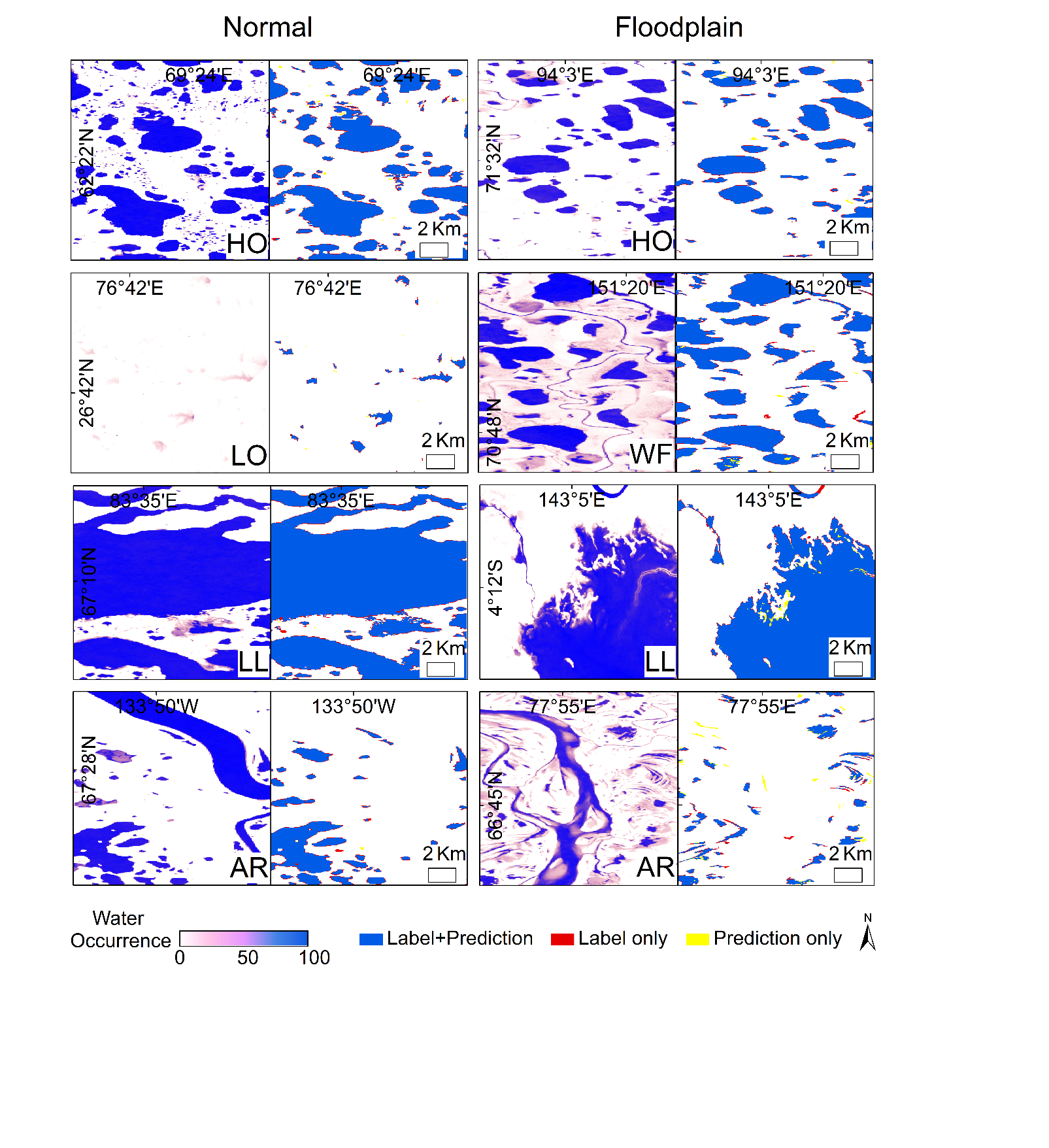
**

## Supplementary Figure 3 | Image pairs revealing the performance of the deep-learning algorithm in predicting lake extents using the test set.

The right panels show the predicted lake extents; the correct classification (Label + Prediction), omission errors (Label only), and commission errors (Prediction only) are color-coded. The left panels are the input images sourced from the GSWO dataset and are independent of the labels used for algorithm training and validation. The lower right annotations represent the abbreviations for the five region types: lakes with middle/high occurrence (HO), lakes with low occurrence (LO), large lakes (LL), lakes alongside rivers (AR) and lakes within floodplains (WF). For specific accuracy statistics, please refer to Supplementary Figure 2 and Supplementary Table 1.

**
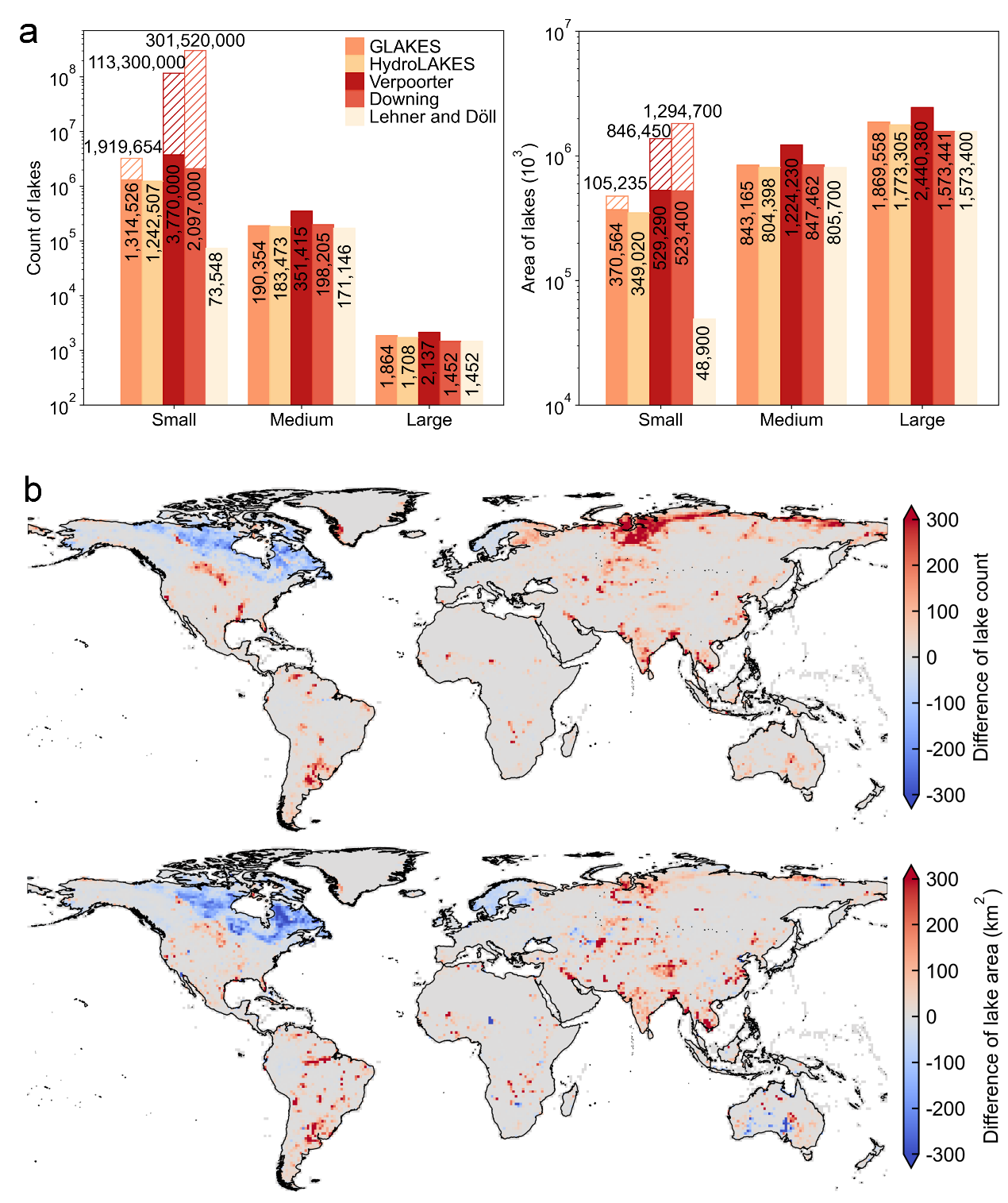
**

## Supplementary Figure 4 | Comparisons of our GLAKES dataset with several previously developed global lake datasets ^1-4^.

**a** Comparison of lake numbers (left) and total area within lake boundaries (right) among different datasets for the three lake size groups. The hatched areas in the small lake group represent the statistics derived for lakes with surface area <0.1 km^2^. **b** The spatial patterns of the differences in lake count and the bounded area between the GLAKES dataset and the HydroLAKES dataset.


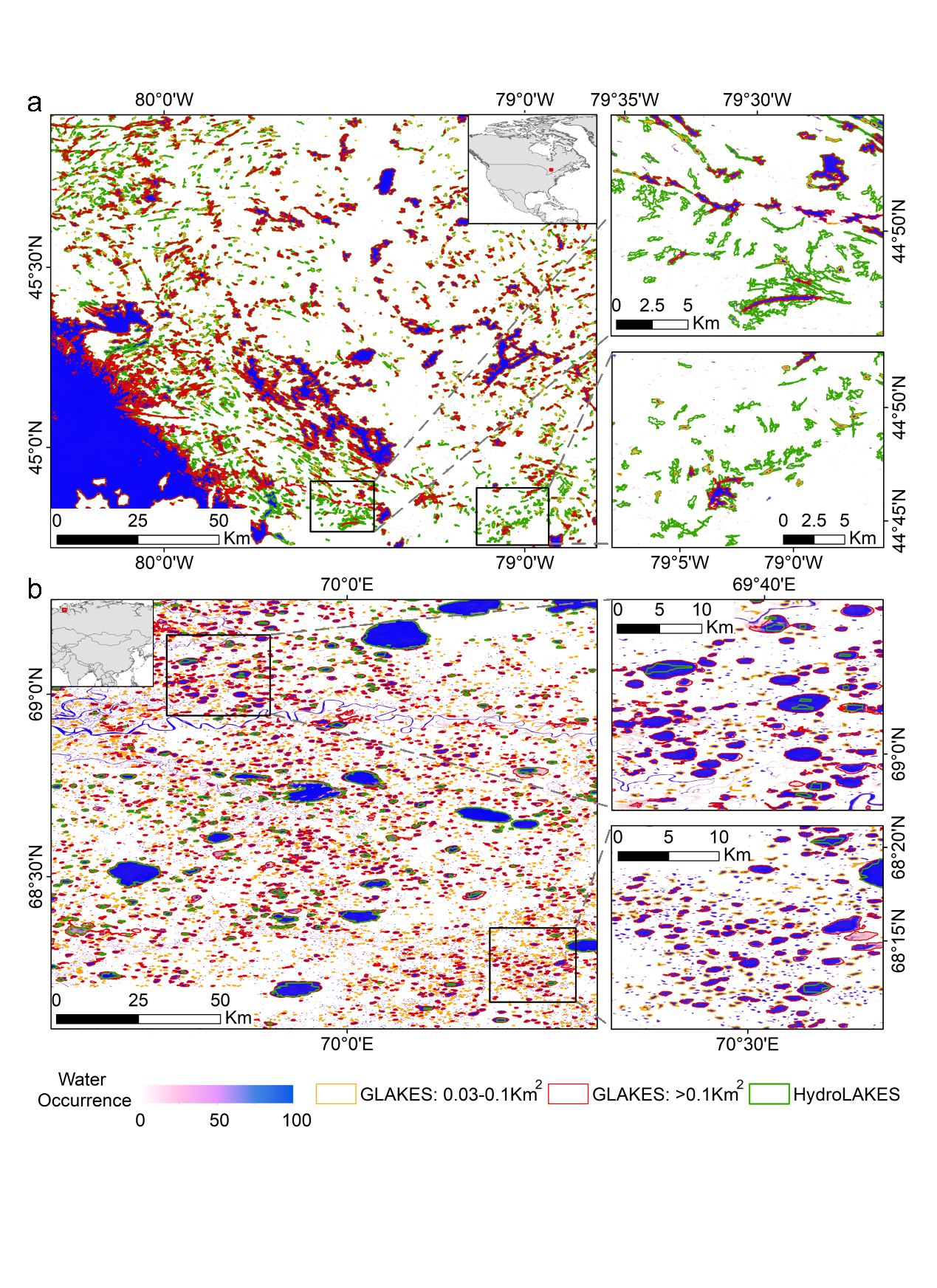


## Supplementary Figure 5 | Examples showing the discrepancies in lake extents between our GLAKES dataset and the HydroLAKES dataset.

**a** Inconsistent delineation of lake extents in eastern Canada, where many regions within the lake boundaries indicated by HydroLAKES show low (or zero) water occurrence, resulting in no detection in the GLAKES dataset. **b** Divergent lake mapping in northern Russia, where many water bodies with high water occurrence (>90%) are not included in HydroLAKES. In particular, a substantial number of lakes with surface area >0.03 km^2^ and <0.1 km^2^ (i.e., the lower limit for HydroLAKES) are mapped in the GLAKES datasets. The background images are obtained from the GSWO dataset.


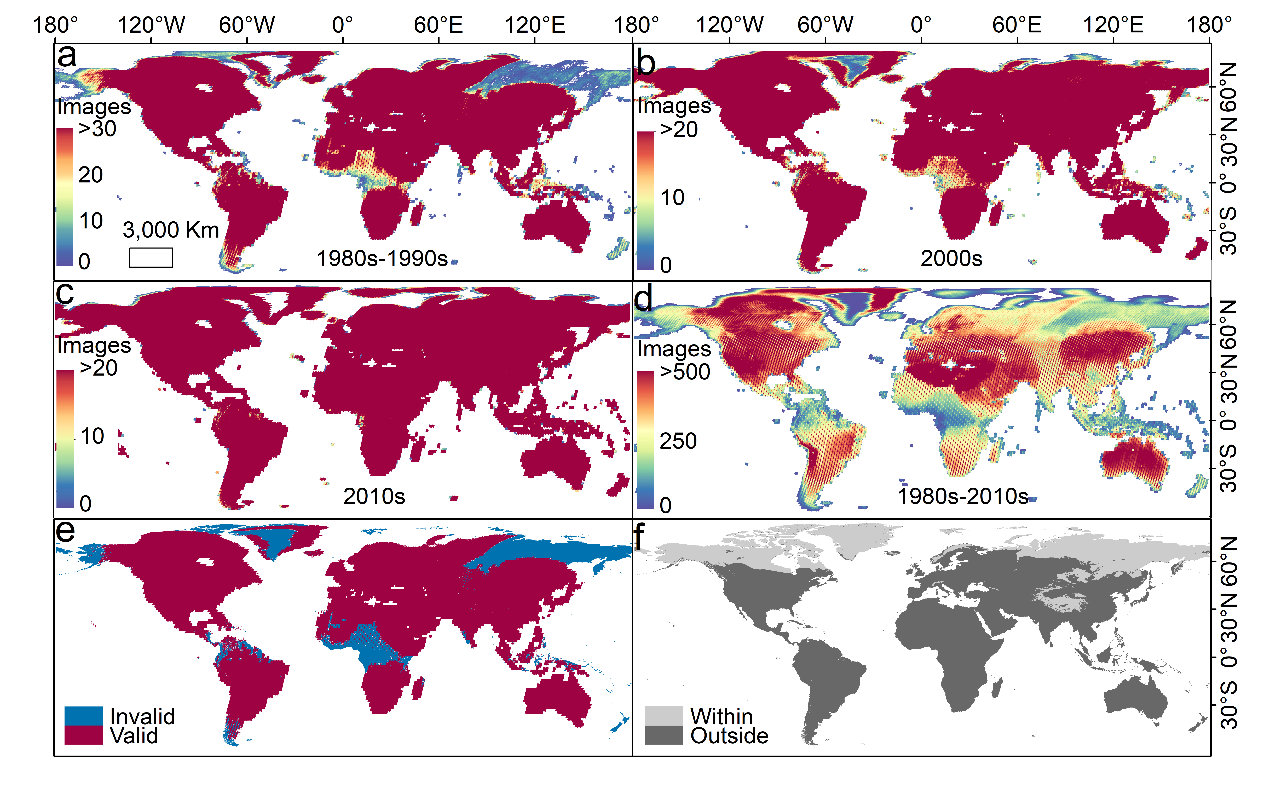


## Supplementary Figure 6 | Coverage of valid Landsat observations used to determine water occurrence in different periods.

Number of valid images used for each period: **a** 1980-1990s, **b** 2000s, **c** 2010s and **d** 1984-2019. **e** Regions with insufficient satellite coverage in early periods (shaded as invalid), which are excluded from the cross-period comparisons. **f** Mask used to identify lakes within (gray) and outside (black) glacier buffers or permafrost regions (see Methods).


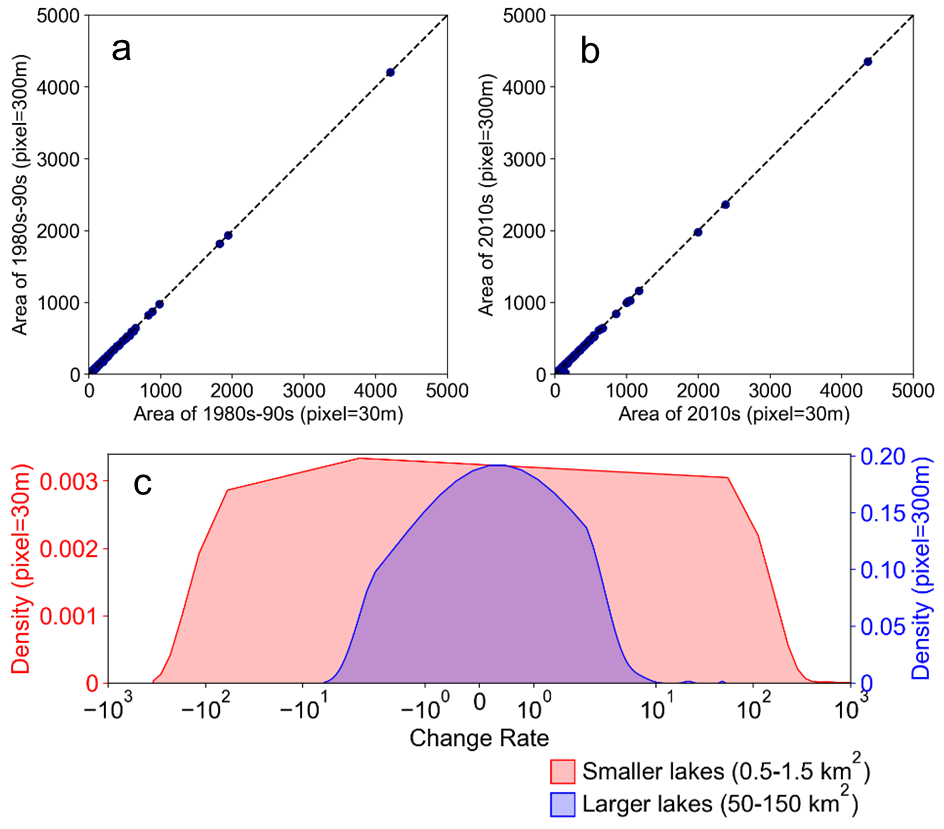


## Supplementary Figure 7 | The impacts of size scale on the estimation of inundation area and relative area changes of lakes in Tibetan Plateau.

Comparison of estimated probability-weighted lake area at different spatial resolutions of the occurrence map in Tibetan Plateau, while **a** is presented for the period 1984-1999 and **b** is for 2010-2019. **c** Comparison of the relative area change rate distribution for lakes in the Tibetan Plateau within the size range of 0.5-1.5 km^2^ at the resolution of 30m (red) vs. those between 50-150 km^2^ at the 300m resolution scale (purple). Note that the total area of the histogram equals 1 for both the blue and purple sides. The range of change rates for smaller lakes (0.5-1.5 km^2^) is larger than that of larger lakes (50-150 km^2^) even when evaluating under the equal relative pixel scale (30m vs. 300m).


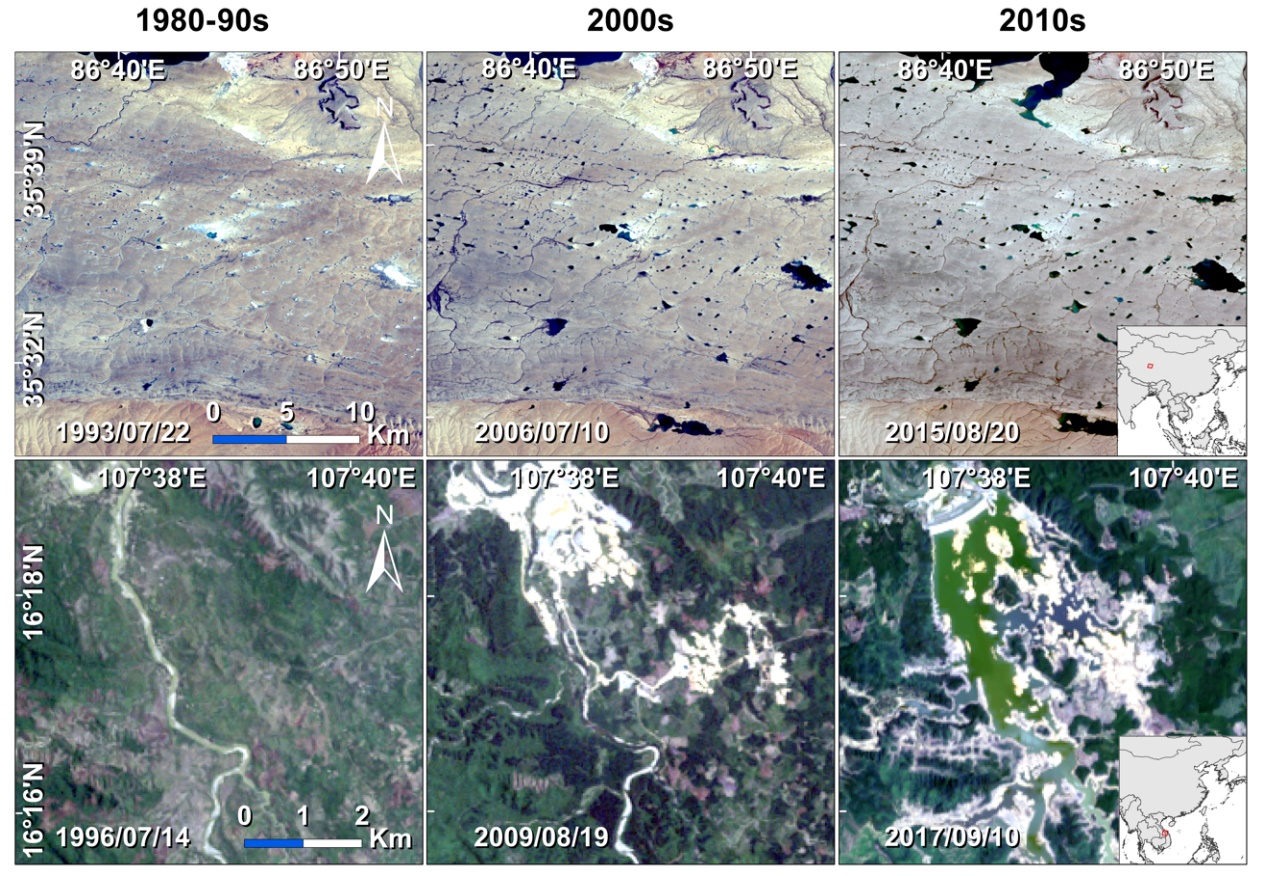


## Supplementary Figure 8 | Examples showing consistently expanding lakes from the 1980-90s to the 2010s.

The upper panels show substantially increased lake area in the Tibetan Plateau, and the lower panels illustrate the impoundment-induced expansion of a reservoir located in Vietnam.


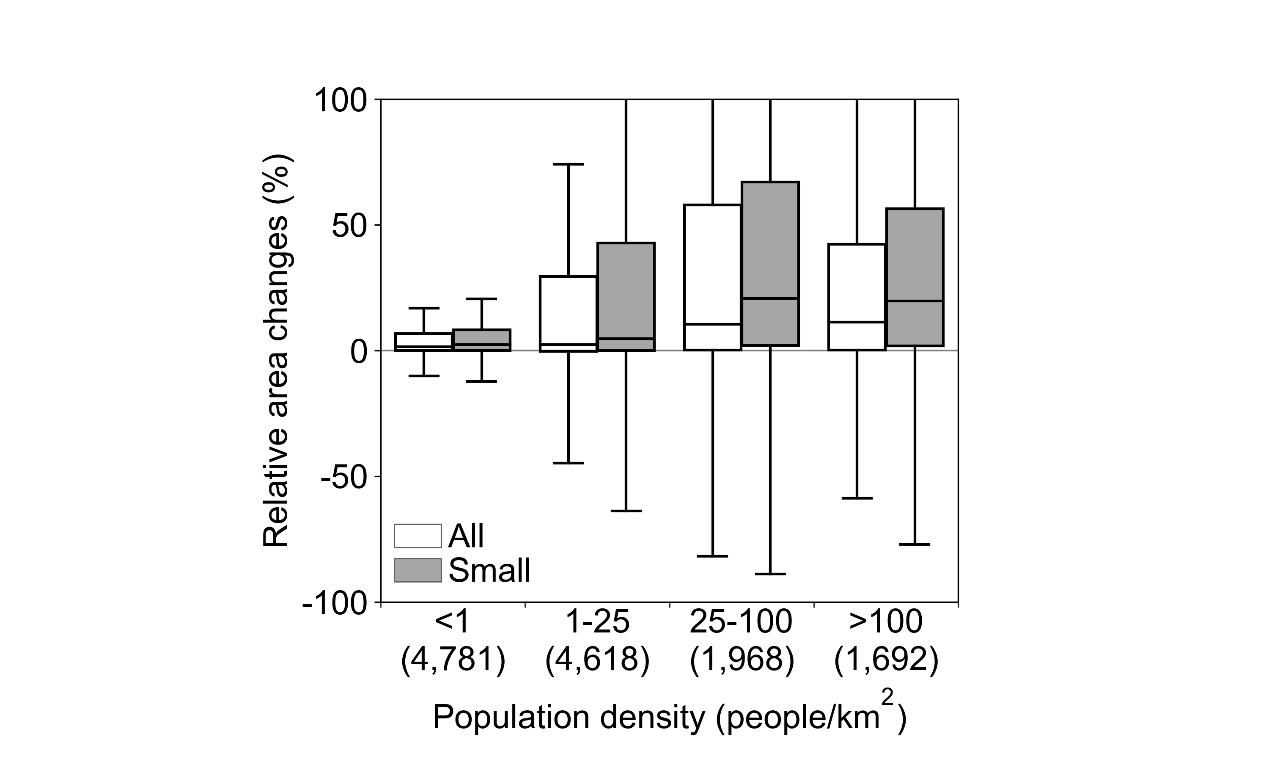


## Supplementary Figure 9 | Box plots of the relative area changes from the 1980-90s to the 2010s grouped into different population densities.

The relative area changes are aggregated values derived from 1°×1° grid cells (similar to Figure 2), and the number of grid cells in each population density group is shown in parentheses. Plots are presented for both the small lake group and for all lakes that are used for grid scale aggregation. Note that the boxplot edges indicate the first (Q_1_) and third quartile (Q_3_) of the data, while the length of whiskers is 1.5 times the IQR (Interquartile range, defined as Q_3_ – Q_1_).


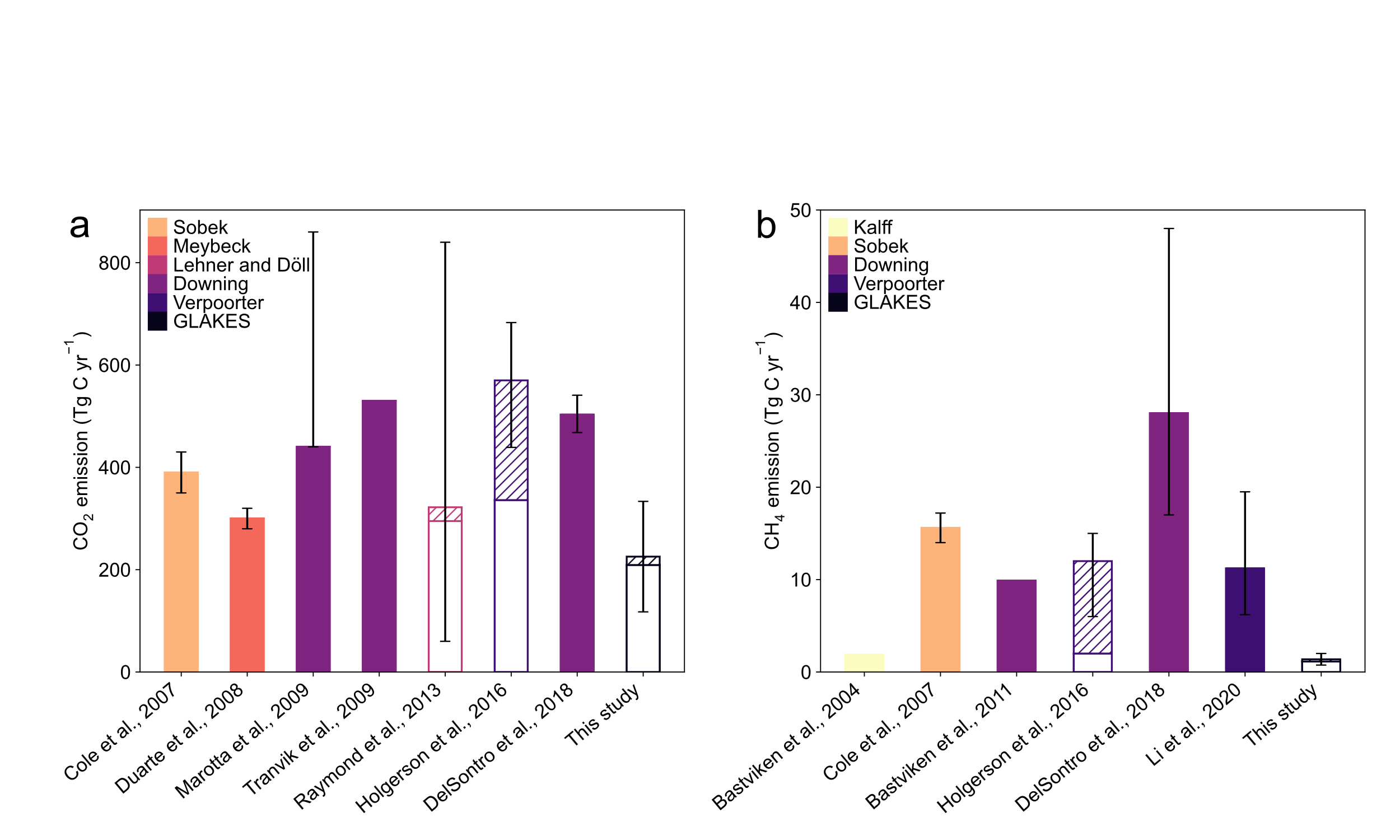


## Supplementary Figure 10 | Comparisons of global lacustrine carbon emissions from Holgerson et al., 2016 with other previous estimates ^5-14^, and the estimated results by applying Holgerson’s method in our GLAKES lake dataset (this study).

**a** CO_2_. **b** CH_4_. Note: 1) Estimations using different lake area datasets ^1-3,15-17^ are marked by different colors. 2) The hatched and empty areas represent the statistics derived for lakes with surface area below and above 0.1 km^2^, where 0.1 km^2^ can be used to define the common size range without extrapolation for all lake datasets listed above. The filled areas denote results that are incapable of subdivision since the original studies did not provide relevant binned size information. 3) The minimum lake size threshold for Holgerson et al., 2016 is 0.0001 km^2^, for Li et al., 2020 is 0.002 km^2^, for Bastviken et al., 2004 & Cole et al., 2007 & Duarte et al., 2008 is unknown, for this study is 0.03 km^2^ and for the others is 0.001 km^2^. 4) The error bars, if any, represent the lower and upper bounds of the estimations, although with different implications. Cole et al., 2007 & Marrotta et al., 2009: min-max; Raymond et al., 2013 & DelSontro et al., 2018: 95% confidence interval; Holgerson et al., 2016 & this study: 25–75th percentiles; The others: unknown. 5) The emissions from both natural lakes and reservoirs are included in most studies except for Duarte et al., 2008, Marotta et al., 2009, Tranvik et al., 2009 and Bastviken et al., 2004, where reservoirs are excluded.

**
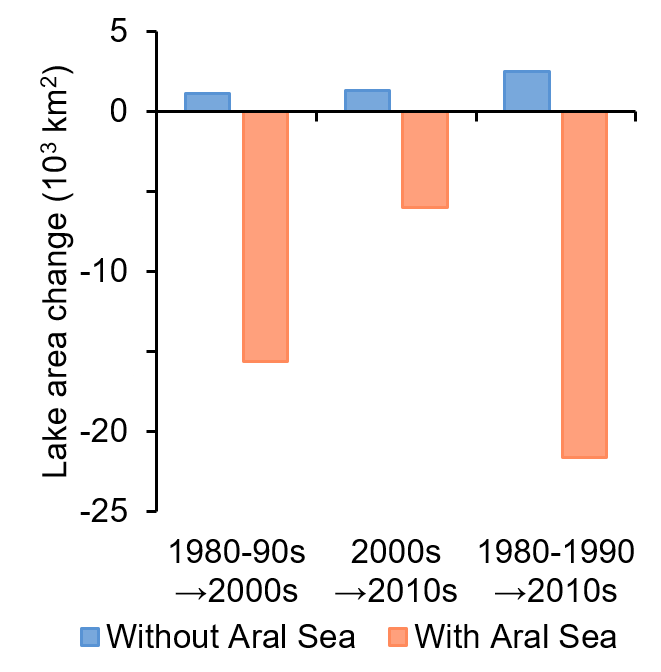
**

## Supplementary Figure 11 | Net area changes in endorheic basins.

The opposite trends can be identified with and without the inclusion of the Aral Sea.

**
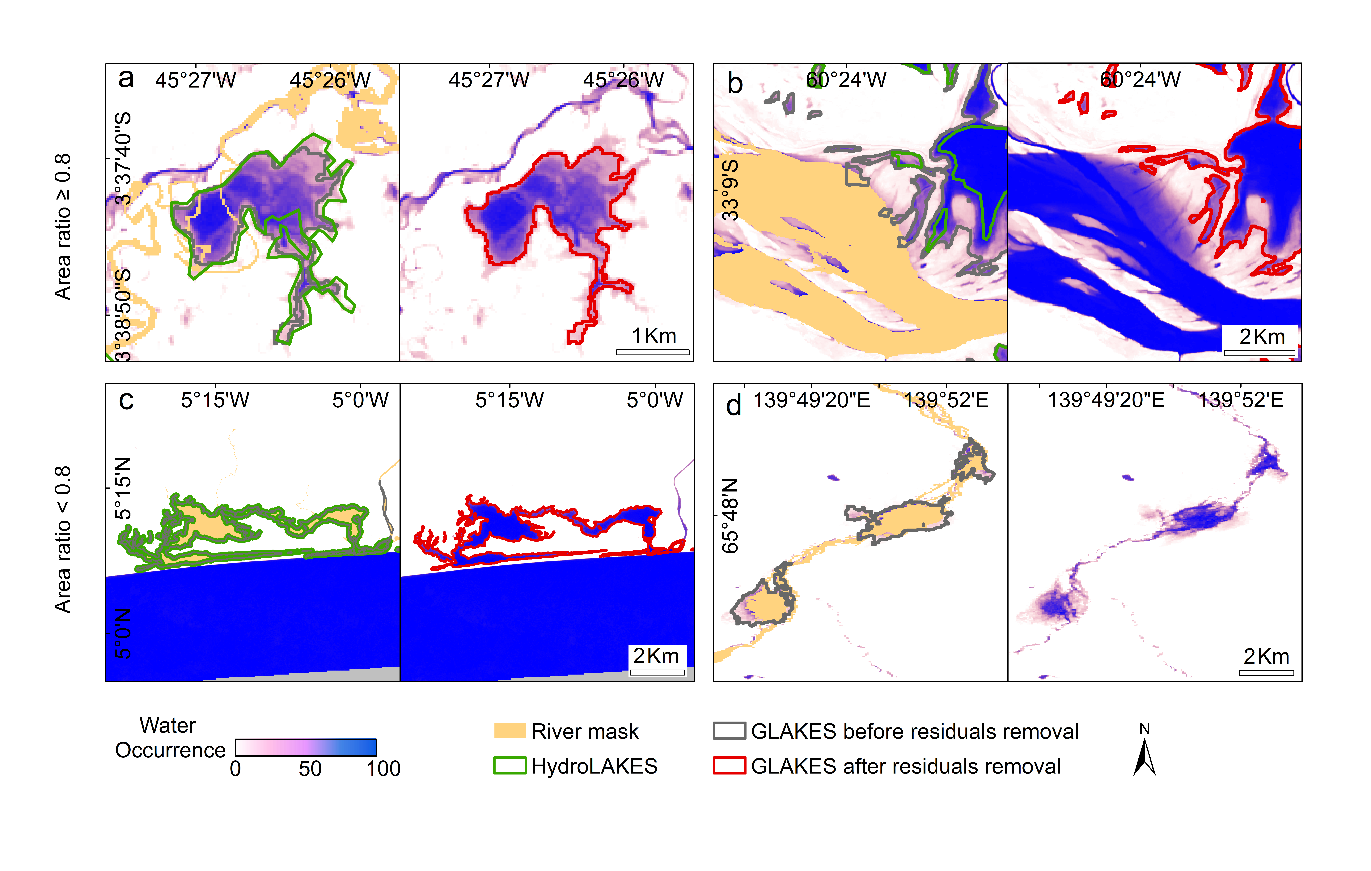
**

## Supplementary Figure 12 | Post-processing of river residual removal and the corresponding results.

**a** Target GLAKES polygons covered by HydroLAKES polygons with an area ratio ≥0.8; **b** Target GLAKES polygons not covered by HydroLAKES polygons with an area ratio ≥0.8; **c** Target GLAKES polygons covered by HydroLAKES polygons with an area ratio <0.8; **d** Target GLAKES polygons not covered by HydroLAKES polygons with an area ratio <0.8. For **a**-**d**, the left figures represent the GLAKES polygons before residuals removal and how they spatially overlay with river masks (GRWL/OSMWL), while the right figures indicate the results after the residual removal post-processing procedure by utilizing the area ratio before/after river masks and the spatial relationship with HydroLAKES.


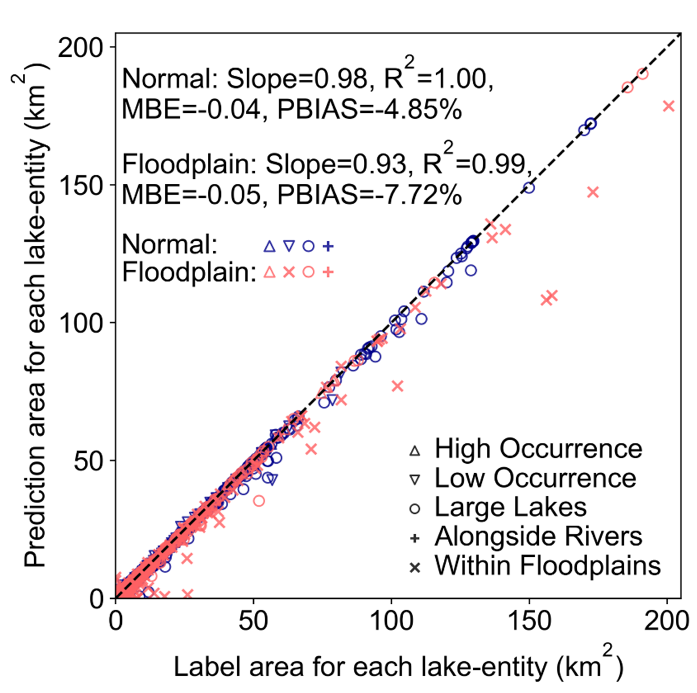


## Supplementary Figure 13 | Validation of the deep-learning algorithm at the lake-entity level.

The predicted area for each lake polygon is compared against the corresponding label area at the lake entity level. Region types are also annotated.


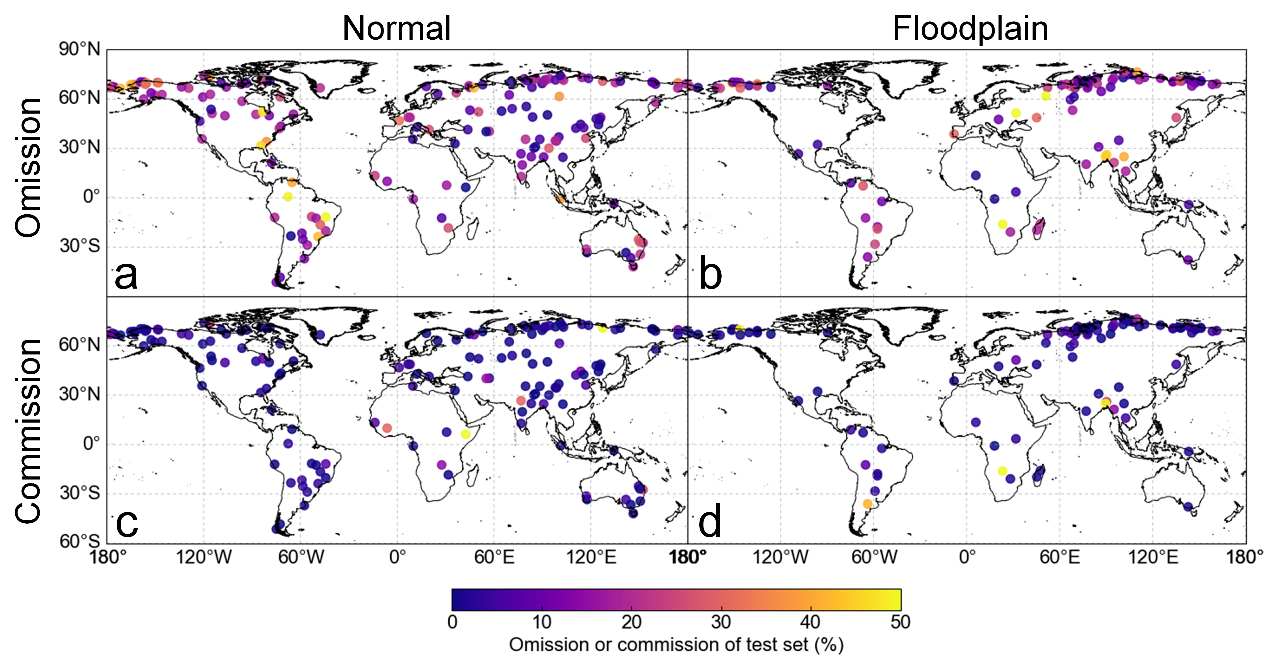


## Supplementary Figure 14 | Validation of the deep-learning algorithm across the globe.

Accuracy assessments (omission/commission errors) for each sample region across the globe based on independent test labels, where the Normal Model and the Floodplain Model are evaluated separately.

# Supplementary Tables

## Supplementary Table 1 | Accuracy assessments of our developed deep-learning algorithm at different lake-size classes.

Error matrix for the GLAKES dataset estimated using independent test labels for the Normal Model and Floodplain Model, listing the accuracy levels derived for different lake size groups.

|  | Size | Omission (%) | Commission (%) | MIoU (%) | Overall Accuracy (%) |
| --- | --- | --- | --- | --- | --- |
| Normal | Small (0.03-1 km^2^) | 23.5 | 2.5 | 94.0 | 99.3 |
|  | Medium (1-100 km^2^) | 4.2 | 0.4 |  |  |
|  | Large (>100 km^2^) | 1.1 | 0 |  |  |
|  | All | 5.4 | 0.5 |  |  |
| Floodplain | Small (0.03-1 km^2^) | 21.2 | 5.0 | 88.7 | 98.7 |
|  | Medium (1-100 km^2^) | 7.3 | 1.7 |  |  |
|  | Large (>100 km^2^) | 9.4 | 0.2 |  |  |
|  | All | 9.6 | 1.9 |  |  |

## Supplementary Table 2 | Keep hyperparameters tested and adjusted in the U-Net Model.

The set of hyperparameter values applied for the final model is also presented.

| Hyperparameters | Setting |
| --- | --- |
| Optimizer | Adadelta |
| Loss function | Tversky: $\alpha$ = 0.5, $\beta$ = 0.5 |
| Batch size | 16 |
| Iteration | Normal model: 750;  Floodplain model: 600 |
| Epoch | 250 |
| Patch size | 512 × 512 |

## Supplementary Table 3 | Accuracy assessments of our developed deep-learning algorithm at the lake-entity level for lakes with different size classes.

Error matrix for the GLAKES dataset estimated using independent test labels for the Normal Model and Floodplain Model, listing the accuracy levels derived for different lake size groups at the lake-entity level. The omission and commission values presented below represent the average of the corresponding values of all lake polygons being assessed.

|  | Size | Omission (%) | Commission (%) |
| --- | --- | --- | --- |
| Normal | Small (0.03-1 km^2^) | 35.0 | 3.0 |
|  | Medium (1-100 km^2^) | 8.3 | 1.0 |
|  | Large (>100 km^2^) | 1.2 | 0 |
|  | All | 32.6 | 2.8 |
| Floodplain | Small (0.03-1 km^2^) | 32.3 | 5.7 |
|  | Medium (1-100 km^2^) | 10.6 | 3.4 |
|  | Large (>100 km^2^) | 9.2 | 0.2 |
|  | All | 30.4 | 5.5 |

## Supplementary Table 4 | A further investigation of the accuracy of our developed deep-learning algorithm in subdivided small lake groups.

Error matrix for the GLAKES dataset estimated using independent test labels for the Normal Model and Floodplain Model, listing the accuracy levels derived for subdivided size groups of small lakes. Both pixel-based and polygon-based results are included for assessments. Likewise, the polygon-based omission and commission values presented below represent the average of the corresponding values of all lake polygons being assessed.

|  | Size | Pixel-based | | Polygon-based | |
| --- | --- | --- | --- | --- | --- |
|  |  | Omission (%) | Commission (%) | Omission (%) | Commission (%) |
| Normal | <0.01 km^2^ | 85.3 | 76.6 | 91.4 | 88.1 |
|  | 0.01-0.03 km^2^ | 52.6 | 17.4 | 54.9 | 22.3 |
|  | 0.03-0.05 km^2^ | 50.5 | 3.6 | 51.5 | 3.7 |
|  | 0.05-0.1 km^2^ | 34.5 | 3.1 | 35.0 | 3.0 |
|  | 0.1-1 km^2^ | 18.9 | 2.3 | 22.2 | 2.5 |
| Floodplain | <0.01 km^2^ | 81.4 | 73.0 | 88.6 | 86.1 |
|  | 0.01-0.03 km^2^ | 51.4 | 21.1 | 53.7 | 25.2 |
|  | 0.03-0.05 km^2^ | 51.2 | 6.7 | 52.8 | 6.8 |
|  | 0.05-0.1 km^2^ | 31.3 | 6.4 | 32.0 | 6.4 |
|  | 0.1-1 km^2^ | 17.0 | 4.7 | 19.5 | 4.8 |

## Supplementary Table 5 | Accuracy assessments of our developed deep-learning algorithm for different region types.

Error matrix for the GLAKES dataset estimated using independent test labels for the Normal Model and Floodplain Model, listing the accuracy levels derived for different region types. Note that only pixel-based results are presented here as the polygon-based results are largely biased by the prevalence of small lake polygons in almost all region types, and thus cannot reflect the true deviations among different region types.

|  | Type Index | Type Name | Omission (%) | Commission (%) |
| --- | --- | --- | --- | --- |
| Normal | 1 | High Occurrence (HO) | 12.4 | 0.3 |
|  | 2 | Low Occurrence (LO) | 8.5 | 1.6 |
|  | 3 | Large Lakes (LL) | 3.3 | 0.2 |
|  | 4 | Alongside Rivers (AR) | 19.6 | 5.0 |
|  | - | All | 5.4 | 0.5 |
| Floodplain | 1 | High Occurrence (HO) | 8.0 | 1.9 |
|  | 3 | Large Lakes (LL) | 4.2 | 0.3 |
|  | 4 | Alongside Rivers (AR) | 12.6 | 1.9 |
|  | 5 | Within Floodplains (WF) | 12.8 | 2.7 |
|  | - | All | 9.6 | 1.9 |

# Supplementary Notes

## Note 1: Details on the U-Net model

### The specific structure of the U-Net model

The U-Net model comprises two major parts: a contracting path for feature interpretation and a near symmetric expanding path for location identification, leading to a u-shaped architecture that enables pixel-to-pixel classification ^18^. In the contracting path, the input feature map undergoes four repeated blocks for downsampling, consisting of two 3x3 convolution layers (each accomplished with a rectified linear unit (ReLU) activation function), a batch normalization layer and a 2x2 max-pooling layer. Notably, the feature channels double after each downsampling process and then half after each upsampling process in the expanding path. Likewise, the expanding path consists of four comparable blocks for upsampling. The difference is that once a 2x2 up-convolution and batch normalization are conducted on the feature map, a concatenation will be performed with its cropped feature map from the corresponding contracting path, and together they go through two 3x3 convolutions activated by ReLU. Finally, a 1x1 convolution layer is used to produce the final classification map.

### The local normalization method used by Brandt, et al. ^19^.

We applied the same local normalization method from Brandt, et al. ^19^ for each patch. That is, the occurrence raster within all patches was normalized first with the mean and standard deviation of the corresponding sample region, while local normalization was performed on 40% of the patches, where image values within these patches were changed to form a standard normal distribution. This process is essential as the omission and commission errors of lake classification would be impacted by the proportion of the local normalization patches.

## Note 2: further investigation of the model’s performance

### Polygon-based assessment at different size scales

We performed a polygon-based assessment to measure the accuracy at each lake entity. As shown in Supplementary Figure 13, our prediction also agreed well with the label area at the polygon base. Notably, the PBIAS here remained the same as the pixel-based assessment results, considering the implication of PBIAS. In addition, we calculated the omission and commission errors for each pair of lake polygons, and the averaged results are presented in Supplementary Table 3. Overall, we observed generally larger omission errors for both models compared to the pixel-based evaluation, in particular for small lakes where the mean omission errors exceeded 30%. In addition, here the average omission errors for size group “all” were actually biased by the prevalent occurrence of small lakes and thus also appeared high (see Supplementary Table 4 ). On the other hand, the commission errors remained low for both models (< 6% across all size groups), reaffirming the high accuracy of our models in terms of misclassification.

### Performance in small lakes with a finer division of size range

We further examined the error matrix of small lakes with a finer division of size range (Supplementary Table 4). Here the reason why we could obtain results with a size <0.03km^2^ (below our cutting threshold of samples) was because the U-Net model learned features at the patch level, where some lakes with area >0.03km^2^ across multiple patches would be split into smaller pieces that might be below 0.03km^2^ and interpreted by our models. As seen in the table, the omission and commission errors generally decreased as the lake size increased. The errors were high for lakes with a size <0.03 km^2^ in both models, with omission errors of >50% and commission errors of >15%. The accuracy was much higher when considering lakes with a size >0.1 km^2^ (the lower size limit for most global lake datasets), where the corresponding omission and commission values dropped below 20% and 5%, respectively. Nevertheless, although lakes with size ranges of 0.03-0.05 km^2^ still faced high omission issues, the commission errors declined suddenly from >15% to ~5%. In practice, we treasured commission errors more than omission errors to ensure that the detected portion of our GLAKES polygons was generally true and thus could be placed with more confidence in further analysis. Therefore, we kept the size threshold as 0.03 km^2^ to include more lakes in our dataset without much compromise on misclassification.

### Performance among 5 different region types

In addition, we also assessed the model’s performance in the 5 categories of sample regions (Supplementary Figure 2b and Supplementary Table 5). Overall, the accuracy of the former three categories (HO, LO, and LL) was higher than that of the remaining two categories (AR and WF), probably owing to the relatively intricate hydrological conditions of the last two types of regions. Here, the largest omission errors (19.6%) of the Normal Model originated from AR, which probably resulted from the missed detection of oxbow lakes that were hardly distinguishable from rivers. Comparative omission errors were found in the Floodplain Model, i.e., WF (12.8%), where the occurrence patterns were very complicated, and the exact floodplain extents were hard to depict. Note that the deviation of the scatter points representing the region type AR was not evident for either models in Supplementary Figure 2b, although exhibiting large omission errors. This is because their containing lake area was generally small and thus, their scatter points were hidden in the lower-left region of the scatter plot with dense scatter concentration. Similarly, commission errors were relatively low in almost all region types, especially compared to omission errors, confirming the model’s conservative strategy in extent delineation for all region types analyzed.

### The spatial distribution of model performance across the globe

The spatial distribution of the omission and commission errors of each patch in the test set is presented in Supplementary Figure 14 to further explore the accuracy of the models spatially. Overall, the omission errors were generally higher than the commission errors in most places regardless of the model type, with the median omission errors being 15.76% and 14.05% and median commission errors being 1.25% and 1.71% for the Normal Model or Floodplain Model, respectively. Spatially, the high omission errors (>20%) in terms of the Normal Model are mainly distributed in Alaska, Siberia, and the Amazon basin. We also revealed that most of these omissions happed in region type 4 (i.e., AR), the major omission source of the Normal Model. In addition, Alaska and Siberia also contributed the most in terms of the number of high omission patches for the Floodplain Model. High omissions also occurred in several other large river floodplains, such as the Ganges River in India and the LaPlata-Parana River in South America. On the other hand, the commission errors of the majority of test patches (> 90% for both models) were below 10%, while only a few left high commission patches distributed sparsely and irregularly across the globe.

## Note 3: uncertainty and limitation

### Global lake mapping

Our global lake coverage did not include ocean-connected lakes and those beyond the latitude range of the GSWO dataset (i.e., 60°S-80°N). In addition, as illustrated above, the features of lakes and rivers could be well distinguished by the model in most regions. Nevertheless, there were lakes that had similar shapes to rivers but were usually short in length, such as some lakes in the Canadian Shield and oxbow lakes alongside rivers. This may be responsible for the relatively high omissions of the region-type AR. In addition, a variety of reservoirs were actually built upon river channels, which were probably identified as rivers and thus resulted in missed detection. To solve this issue, we replaced the U-Net predictions of the on-river reservoirs (defined by GRWL) with those yielded from the automatic extraction method used in sample preparation.

The disentanglement of lakes from floodplains was also challenging as the definition of floodplain extent was ambiguous and arduous. Here we used a globally uniform threshold (30% occurrence) to depict the lake/floodplain interface alongside rivers, which may cause bias on a regional scale. Besides, in post-processing procedures, the floodplain buffers were restricted by the presence and accuracy of the GRWL layer, leading to negligence of floodplains outside the 1 km river buffers and those resulting from the absence of the underlying GRWL mask. Equally challenging was the division between natural lakes and agricultural fields. First of all, given the inherent constraint of the GSWO dataset, not all paddy fields were mapped ^20^. Besides, the size threshold of 0.03 km^2^ was helpful in screening out some small and isolated agricultural fields. In addition, compared to natural lakes, a vast proportion of agriculture fields exhibit regular shapes, grided textures, and low occurrence in GSWO maps, serving as a basic principle for discrimination from natural lakes. However, since the focus of this study was mainly the partition of lentic and lotic water systems, these human-transformed water bodies were temporarily considered as lakes in our GLAKES dataset.

The setting of a fixed cutting threshold was probably the major reason for the relatively high omission errors for small lakes (especially those around 0.03km^2^). This could be induced by the influence of the negative samples. Specifically, in the process of sample preparation, we applied a filter to screen out all lakes with sizes <0.03km^2^. In fact, the difference between lakes just exceeding the size threshold, and those approximating the thresholds (e.g., 0.031km^2^ and 0.029 km^2^) were probably minor. Therefore, the setting of a fixed cutting threshold for samples may somewhat confuse the model, in a way that some lakes were interpreted as true lakes (because they were marked by our labels) while the others with similar features may be identified by the model as non-lakes (due to the lack of overlaying labels), thus leading to a certain extent of missed detections of small lakes around the size of the cutting threshold (0.03 km^2^). Under such circumstances, a better solution may be the inclusion of samples with size <0.03 km^2^ in model training, followed by a result-oriented determination of the cutting threshold for lake predictions (i.e., finding out a threshold where the accuracy for lakes below the certain size threshold was unacceptable, if possible).

Uncertainty and limitation could also result from the auxiliary dataset, such as the river mask and water occurrence map used for lake mapping. According to the developing procedure of the GSWO map, only waters that were visible from space (i.e., Landsat observation) without any overlaying obstacles were able to be included in the final surface water mapping. This could lead to missed detection of frozen lakes and vegetated wetlands and thus an underestimation of lake coverage in regions like the Canadian Shield and Scandinavia. In addition, it could be observed that inconsistencies between the GSW occurrence map and the GRWL and OSMWL river masks existed in many regions. On the one hand, the coverage of GRWL and OSMWL for many large rivers was inadequate compared to that of the occurrence map. Application of these river masks for river exclusion would lead to a large number of river residuals that required further elimination (especially through manual revision). On the other hand, a small percent of lakes also had the risk of being masked mistakenly due to such inconsistencies. Nevertheless, the overall accuracy of the utilized river masks was generally satisfactory, and the impact of dataset inconsistency could be reduced by using the area ratio before and after- the river mask, as mentioned above.

The capability of the U-Net models in differentiating large rivers with broad river widths from lakes would be constrained by the scale of the patches that served as the basic unit for feature learning. An enlargement of the patch size could partially solve this problem but would greatly increase the GPU memory as well. The 512 x 512 pixel was the largest scale that was applicable in our study, given our maximum GPU RAM of 24 GB. Hence, we utilized auxiliary river masks in label generation as well as the post-processing process for exclusion of the remaining rivers, although this introduced new uncertainty as stated before.

### Temporal change in probability-weighted lake area

In temporal change analysis, owing to the constraint of the valid observations of Landsat images, this study mainly focused on the changes in lake extent at the decadal scale. However, lake dynamics at shorter timescales could also be evident. Pickens, et al. ^21^ discovered that only 23% of the total area of open surface water was permanent without ice cover within 2019, while permanent water covered by seasonal ice/snow constituted 41% of the total area, and the remaining 36% was made up of seasonal waters regardless of ice coverage. Furthermore, such seasonal patterns of water/land/ice transition may undergo substantial changes during the whole study period owing to the impact of climate change, where the ice cover duration experienced intensifying reductions and the wetting/drying trends variated in different regions ^22-26^. These changes at a shorter timescale may impose divergent impacts on our decadal change analysis of lake surface area. The impacts of seasonal water/land transition along with its trend were minimal as they have already been incorporated into the occurrence map and thus the calculation of probability-weighted lake area each period. However, the negligence of lake ice coverage (in the GSW MWH dataset, ice was flagged as invalid observations) might lead to a conservative extraction of the lake outlines as well as an underestimation of the water occurrence value. Given that the extension of the ice-free season was reported to exhibit an increasing trend ^26,27^, the underestimation of probability-weighted lake area might be less severe in the more recent period, indicating that there might be a slight overestimation of the calculated lake area changes over different periods in places covered with ice.

### Temporal change in carbon emissions

In this study, the method we used had already been applied in a previous study to calculate lacustrine carbon emissions ^12^. It was calculated by multiplying the average emission rate (flux) of different lake size classes by the total lake area accounting for corresponding size bins. However, the average emission rates were aggregated from finite in-situ measurements that may not reflect the true global distribution ^13,28^, which limited the accuracy of our global estimates. In particular, the empirical size-dependent flux values listed in Holgerson et al. ^12^ were derived from in situ lake samples with certain geographic dependencies (e.g., the majority of sampled lakes were located within the range of 30°N-70°N), which may cause uncertainty when upscaling for global lakes. Besides, the incorporation of other relevant drivers such as the water body type, water depth, water productivity, sediments as well as ecoclimate zone would also enhance the representativeness of the average emission rates for more accurate global estimates ^13,29-31^. Using the water body type as an example, here we combined the total area of reservoirs and natural lakes to obtain the global estimation results, without more discrete classification, as in several other studies ^11,13,14^. Nevertheless, previous studies have revealed that methane emissions in different water body types might be driven by different processes, which would inevitably impact the final estimation results ^31-33^.

In addition, in calculating the changes over three periods, we kept the average flux values constant, considering only the long-term carbon emission changes that were brought by the lake area variations over different time periods. Nevertheless, the transfer of carbon gases from the aquatic environment to the atmosphere is a highly dynamic process, which could also be modulated by lake dynamics (such as ice phenology and water/land transitions) at shorter timescales ^12,13,30,32,34,35^. It has been reported that CO_2_ and CH_4_ accumulate under the ice, and subsequently vent a substantial amount to the atmosphere during the spring melt, during which CH_4_ oxidation may co-occur, although this is probably not applicable to oligotrophic lakes or completely frozen lakes ^5,36-38^. Considering the trend of global warming over the study period, the ice-free seasons for most lakes extended, and some permanently frozen lakes became seasonally ice-covered, leading to a further boost to global carbon emissions ^23,30,39^. Besides, the seasonal drying and wetting of lakes was also an important carbon emission source. Studies have revealed complex relationships between water level and aquatic carbon emissions and identified dry aquatic sediments as significant carbon gas hot spots ^34,35,40,41^, which we also did not account for. Given the variated wetting/drying trends across different regions globally ^24-26^, the overall impact on long-term carbon emission changes is hard to quantify, and more data are required for systematic evaluations.

While our study only paid attention to the diffusive CH_4_ flux, CH_4_ ebullition was deemed the dominant CH_4_ emission pathway ^5^. However, the data availability of direct in-situ ebullition rate measurements and their high spatiotemporal variability hampers the systematic assessments of CH_4_ ebullition at a global scale ^12,42^. Nevertheless, although the accuracy of our global-scale carbon emission estimates would be impacted by the abovementioned factors, the main objective of this section is to highlight the essential roles of small lakes in driving lacustrine carbon emissions, which remain robust.

# Supplementary References

1 Lehner, B. & Döll, P. Development and validation of a global database of lakes, reservoirs and wetlands. *Journal of hydrology* **296**, 1-22 (2004).

2 Downing, J. A. *et al.* The global abundance and size distribution of lakes, ponds, and impoundments. *Limnology and Oceanography* **51**, 2388-2397 (2006).

3 Verpoorter, C., Kutser, T., Seekell, D. A. & Tranvik, L. J. A global inventory of lakes based on high‐resolution satellite imagery. *Geophysical Research Letters* **41**, 6396-6402 (2014).

4 Messager, M. L., Lehner, B., Grill, G., Nedeva, I. & Schmitt, O. Estimating the volume and age of water stored in global lakes using a geo-statistical approach. *Nature communications* **7**, 1-11 (2016).

5 Bastviken, D., Cole, J., Pace, M. & Tranvik, L. Methane emissions from lakes: Dependence of lake characteristics, two regional assessments, and a global estimate. *Global biogeochemical cycles* **18** (2004).

6 Cole, J. J. *et al.* Plumbing the global carbon cycle: integrating inland waters into the terrestrial carbon budget. *Ecosystems* **10**, 172-185 (2007).

7 Duarte, C. M. *et al.* CO2 emissions from saline lakes: A global estimate of a surprisingly large flux. *Journal of Geophysical Research: Biogeosciences* **113** (2008).

8 Marotta, H., Duarte, C. M., Sobek, S. & Enrich‐Prast, A. Large CO2 disequilibria in tropical lakes. *Global biogeochemical cycles* **23** (2009).

9 Tranvik, L. J. *et al.* Lakes and reservoirs as regulators of carbon cycling and climate. *Limnology and oceanography* **54**, 2298-2314 (2009).

10 Bastviken, D., Tranvik, L. J., Downing, J. A., Crill, P. M. & Enrich-Prast, A. Freshwater methane emissions offset the continental carbon sink. *Science* **331**, 50-50 (2011).

11 Raymond, P. A. *et al.* Global carbon dioxide emissions from inland waters. *Nature* **503**, 355-359 (2013).

12 Holgerson, M. A. & Raymond, P. A. Large contribution to inland water CO 2 and CH 4 emissions from very small ponds. *Nature Geoscience* **9**, 222-226 (2016).

13 DelSontro, T., Beaulieu, J. J. & Downing, J. A. Greenhouse gas emissions from lakes and impoundments: Upscaling in the face of global change. *Limnology and Oceanography Letters* **3**, 64-75 (2018).

14 Li, M. *et al.* The significant contribution of lake depth in regulating global lake diffusive methane emissions. *Water research* **172**, 115465 (2020).

15 Meybeck, M. in *Physics and chemistry of lakes* 1-35 (Springer, 1995).

16 Jacob, K. *Limnology: Inland Water Ecosystems 2nd Edition. Prentice Hall, Upper Saddle River.*, (2002).

17 Sobek, S., Tranvik, L. J. & Cole, J. J. Temperature independence of carbon dioxide supersaturation in global lakes. *Global Biogeochemical Cycles* **19** (2005).

18 Ronneberger, O., Fischer, P. & Brox, T. U-net: Convolutional networks for biomedical image segmentation. *International Conference on Medical image computing and computer-assisted intervention*, 234-241 (2015).

19 Brandt, M. *et al.* An unexpectedly large count of trees in the West African Sahara and Sahel. *Nature* **587**, 78-82 (2020).

20 Pekel, J.-F., Cottam, A., Gorelick, N. & Belward, A. S. High-resolution mapping of global surface water and its long-term changes. *Nature* **540**, 418-422 (2016).

21 Pickens, A. H. *et al.* Global seasonal dynamics of inland open water and ice. *Remote Sensing of Environment* **272**, 112963 (2022).

22 Magnuson, J. J. *et al.* Historical trends in lake and river ice cover in the Northern Hemisphere. *Science* **289**, 1743-1746 (2000).

23 Sharma, S. *et al.* Widespread loss of lake ice around the Northern Hemisphere in a warming world. *Nature Climate Change* **9**, 227-231 (2019).

24 Greve, P. *et al.* Global assessment of trends in wetting and drying over land. *Nature geoscience* **7**, 716-721 (2014).

25 Roderick, M., Sun, F., Lim, W. H. & Farquhar, G. A general framework for understanding the response of the water cycle to global warming over land and ocean. *Hydrology and Earth System Sciences* **18**, 1575-1589 (2014).

26 Woolway, R. I. *et al.* Global lake responses to climate change. *Nature Reviews Earth & Environment* **1**, 388-403 (2020).

27 Wang, X. *et al.* Continuous loss of global lake ice across two centuries revealed by satellite observations and numerical modeling. *Geophysical Research Letters*, e2022GL099022 (2022).

28 Rosentreter, J. A. *et al.* Half of global methane emissions come from highly variable aquatic ecosystem sources. *Nature Geoscience* **14**, 225-230 (2021).

29 Rasilo, T., Prairie, Y. T. & Del Giorgio, P. A. Large‐scale patterns in summer diffusive CH 4 fluxes across boreal lakes, and contribution to diffusive C emissions. *Global Change Biology* **21**, 1124-1139 (2015).

30 Wik, M., Varner, R. K., Anthony, K. W., MacIntyre, S. & Bastviken, D. Climate-sensitive northern lakes and ponds are critical components of methane release. *Nature Geoscience* **9**, 99-105 (2016).

31 Deemer, B. & Holgerson, M. A. Drivers of methane flux differ between lakes and reservoirs, complicating global upscaling efforts. *Journal of Geophysical Research: Biogeosciences* **126**, e2019JG005600 (2021).

32 Deemer, B. R. *et al.* Greenhouse gas emissions from reservoir water surfaces: a new global synthesis. *BioScience* **66**, 949-964 (2016).

33 Hayes, N. M., Deemer, B. R., Corman, J. R., Razavi, N. R. & Strock, K. E. Key differences between lakes and reservoirs modify climate signals: A case for a new conceptual model. *Limnology and Oceanography Letters* **2**, 47-62 (2017).

34 Chamberlain, S. D. *et al.* Influence of transient flooding on methane fluxes from subtropical pastures. *Journal of Geophysical Research: Biogeosciences* **121**, 965-977 (2016).

35 Keller, P. S., Marcé, R., Obrador, B. & Koschorreck, M. Global carbon budget of reservoirs is overturned by the quantification of drawdown areas. *Nature Geoscience* **14**, 402-408 (2021).

36 Kortelainen, P. *et al.* Sediment respiration and lake trophic state are important predictors of large CO2 evasion from small boreal lakes. *Global Change Biology* **12**, 1554-1567 (2006).

37 Michmerhuizen, C. M., Striegl, R. G. & McDonald, M. E. Potential methane emission from north‐temperate lakes following ice melt. *Limnology and Oceanography* **41**, 985-991 (1996).

38 Utsumi, M. *et al.* Dynamics of dissolved methane and methane oxidation in dimictic Lake Nojiri during winter. *Limnology and Oceanography* **43**, 10-17 (1998).

39 Natchimuthu, S., Panneer Selvam, B. & Bastviken, D. Influence of weather variables on methane and carbon dioxide flux from a shallow pond. *Biogeochemistry* **119**, 403-413 (2014).

40 Marcé, R. *et al.* Emissions from dry inland waters are a blind spot in the global carbon cycle. *Earth-science reviews* **188**, 240-248 (2019).

41 Tangen, B. A. & Bansal, S. Hydrologic lag effects on wetland greenhouse gas fluxes. *Atmosphere* **10**, 269 (2019).

42 Beaulieu, J. J., McManus, M. G. & Nietch, C. T. Estimates of reservoir methane emissions based on a spatially balanced probabilistic‐survey. *Limnology and Oceanography* **61**, S27-S40 (2016).
